# Mycorrhizal extraradical mycelium can reduce cadmium uptake by maize and cadmium leaching from contaminated soil: based on an in-growth core experiment

**DOI:** 10.3389/fmicb.2024.1507798

**Published:** 2024-12-16

**Authors:** Yijie Yang, Yang Li, Xiaoyi Li, Jie Yan, Longhua Wu, Zhenwu Tang, Yongmei He, Fangdong Zhan

**Affiliations:** ^1^College of Resources and Environment, Yunnan Agricultural University, Kunming, China; ^2^Nanjing Institute of Soil Science, Chinese Academy of Sciences, Nanjing, China; ^3^College of Resources and Environment, Southwest University, Chongqing, China

**Keywords:** arbuscular mycorrhizal fungi, extraradical mycelium, ingrowth core, soil solution, cadmium form, leaching loss

## Abstract

Arbuscular mycorrhizal fungi (AMF) are commonly found in heavy metal-contaminated environments and form extraradical mycelium (ERM), but knowledge of their ecological functions is limited. In the present study, a soil column was filled with sterilized cadmium (Cd)-contaminated soil and contained an in-growth core for AMF-inoculated maize seedling growth. The in-growth core was static to maintain or rotated to disrupt ERM growth. Compared with the static treatment, the rotation treatment caused significant decreases in the content of glomalin-related soil protein (GRSP), the photosynthetic physiology of leaves, and maize growth, while increasing the Cd content in shoots and roots by 64 and 82%, respectively; additionally, the rotation treatment resulted in increases in the Cd concentration of the soil solution inside and outside the growth core by 30–38 and 17–52%, respectively, and Cd leaching loss by 29–39%. Moreover, the rotation treatment significantly altered the Cd forms in the soil solution and leachate, increasing the proportion of free Cd^2+^ by 0.8–2.1% and decreasing the proportions of CdSO_4_(aq) and CdOH^+^ by 6.1–56.1% and 26.1–48.7%, respectively. The structural equation model indicated that AMF directly and indirectly reduced maize Cd uptake and Cd leaching loss by decreasing Cd availability in the soil and soil solution through the GRSP secreted by ERM. Thus, AMF plays a crucial role in inhibiting Cd migration in soil through mycelial exudates.

## 1 Introduction

Heavy metal (HM) pollution in soil is currently one of the most prominent environmental problems (Mohammed et al., 2011). According to the National Soil Pollution Survey Bulletin, the area of HM-contaminated farmland accounts for 1/5 of the arable land (Zhao et al., 2015). Moreover, surface runoff and leaching under rainfall or irrigation conditions led to the downward migration of HMs such as cadmium (Cd) that accumulated in the soil surface layer, which in turn triggered Cd leaching loss and pollution diffusion into deeper soil and groundwater (Gray et al., 2017). Cd contamination in soil can induce adverse effects such as soil degradation, reduced fertility, and hindered plant growth and development (Haider et al., 2021).

Cd migration from contaminated soil can be affected by soil microorganisms (NaziaTahir et al., 2021). Arbuscular mycorrhizal fungi (AMF) are crucial soil microorganisms that engage in symbiotic relationships with over 80% of terrestrial plants, offering beneficial effects on plant growth and Cd uptake (Riaz et al., 2021). AMF not only improved the physical and chemical properties of contaminated soil but also promoted nutrient and water uptake by the host plant and promoted plant performance under Cd stress (Kavadia et al., 2020; Ghobadi et al., 2020; Zhan et al., 2019). AMF colonizes plant roots and forms mycorrhizae, which can alleviate Cd toxicity in plants and inhibit Cd uptake by blocking Cd transfer from roots to shoots (Riaz et al., 2021). Moreover, AMF altered root growth, including root length and biomass, and consequently affected Cd transfer and uptake (Guo et al., 2023). Additionally, a large number of mycorrhizal extraradical mycelium (ERM) can bind to Cd ions through mycelial exudates, thus significantly impacting Cd migration in soil (Ferrol et al., 2016). ERM can bind Cd by secreting glomalin-related soil protein, which can increase Cd adsorption in soil and reduce Cd leaching loss (He et al., 2020; Gonzalez-Chavez et al., 2004). However, most of the related research has focused on the effects of AMF on plant growth by improving the uptake of nutrients and water under Cd stress (Yu et al., 2021; Chang et al., 2018). However, the understanding of the effects of ERM and its underlying mechanisms on Cd migration in soil is limited.

Some experimental devices have been developed to investigate ERM function. The in-growth core is a special experimental device consisting of a transparent container or tube that simulates the real growth environment of plants in soil and is usually used to study the effects of ERM on plant growth (Leifheit et al., 2014; Wu et al., 2016). The in-growth core can effectively separate the root and fungal mycelium via microporous mesh with a pore diameter of 20–50 μm to maintain internal root growth and allow the ERM to grow to the outside of the core. Furthermore, the in-growth core remained static to maintain or rotate to disrupt the growth of ERM outside the core. Previous studies revealed that the disruption of ERM by rotation of the in-growth core led to a decrease in the level of glomalin-related soil protein (GRSP) and a significant decrease in the growth and nutrient uptake of maize, suggesting that ERM is critical for plant growth (Wu et al., 2016; Liu et al., 2014).

In the present study, an in-growth core was developed according to the method of Johnson et al. (2001), and it was put into a soil column filled with sterilized Cd-contaminated soil. The in-growth core was static or rotated to maintain or disrupt ERM growth in the soil column. The effects of ERM on maize growth, the Cd concentration and form in the soil solution inside and outside the in-growth core, and the loss of Cd from the soil column were investigated. It was hypothesized that ERM plays a crucial role in inhibiting Cd uptake by plants and Cd leaching loss from soil by reducing the Cd concentration and form in soil solution.

## 2 Materials and methods

### 2.1 Materials

Soil samples were collected from Cd-contaminated farmland near the lead-zinc mining region in Huize County, Qujing City, Yunnan Province, China (latitude 26°31′10″N, longitude 103°34′56″E). After natural air-drying, the soil was sieved through a sieve with a pore diameter of 2 mm and autoclave sterilized at 120°C for 2 h. The physical and chemical properties of the soil are shown in Supplementary Table S1.

The AMF inoculant was *Funneliformis mosseae* (BGC YN05, 1511C0001BGCAM0013), which was provided by the Institute of Plant Nutrition and Resources, Beijing Academy of Agricultural and Forestry Sciences. The AMF inoculant contained approximately 18 spores per gram.

Seeds (variety of Huidan 4) of *Zea mays* L. were used in the present study. After surface disinfection with 75% ethanol (soaking for 1 min) and 10% sodium hypochlorite (soaking for 2 min), the maize seeds were placed in a constant temperature incubator at 28°C for 3 days to germinate into a 0.5–1 cm long seedling.

### 2.2 Experimental device

In the research, a soil column was filled with sterilized Cd-contaminated soil and contained an in-growth core for maize seedling growth (Supplementary Figure S1). The soil column was made of polyvinyl chloride (PVC) with a diameter of 110 mm and a height of 300 mm, and a drainage tap was installed at the bottom to drain the leachate. Within the soil column, an in-growth core with a diameter of 50 mm and a height of 240 mm was produced according to the method of Johnson et al. (2001). There were holes with a diameter of 3 cm around the in-growth core, which was covered by a nylon mesh of 400 mesh (37 μm) that allowed the ERM but prevented the root from passing through. Additionally, samplers were installed inside the in-growth core and the soil column at 10 and 20 cm depths for sampling the soil solution.

### 2.3 Experimental design

The sterilized soil in the in-growth core was planted with a maize seedling and inoculated with 50 g of AMF inoculant. The treatments included static (the in-growth core remained static to maintain ERM growth inside the soil column) and rotated (the in-growth core rotated every 3 days to disrupt ERM growth inside the soil column) treatments. Thus, there were 5 pots per treatment and 10 pots in total.

The experiment was carried out in a greenhouse. After the maize seedlings had grown to a height of 5 cm for 5 days, the in-growth core began to rotate every 3 days, rotating about 45° along the vertical axis to deter mycorrhizal extraradical mycelium (ERM) penetration into the core.

After 30 days of maize seedlings grown, 3 leaching events were conducted every 7 days on Days 31, 38, and 45. The soil column received 3 L of deionized water over 1 h at a drip irrigation rate of 50 mL/min. After irrigation, the soil solution was sampled from the inside and outside of the in-growth core at depths of 10 cm and 20 cm, respectively, in the soil column using a solution sampler (Rhizon 19.21.23F, Environment Purification Science and Technology Limited Company, Beijing, China). The leachate was collected through a drainage tap at the bottom of the soil column. Subsequently, the soil solution and leachate were filtered through a 0.45 μm membrane for subsequent analyses.

### 2.4 Evaluation of maize photosynthesis, height, and biomass traits

After 45 days of maize growth, plant height was measured with steel tape. The photosynthesis of the third leaf at the top of maize, including the photosynthetic rate, intercellular CO_2_ concentration, and transpiration rate, was measured by a portable photosynthesis analyzer (LCpro T, ADC Bioscientific Ltd., Hoddesdon, UK). Fresh leaves (0.5 g) were weighed and placed in a centrifuge tube, ground with 80% ethanol solution to extract the chlorophyll, filtered through filter paper, and transferred to a 25 mL volumetric flask. The absorbance was measured at 645 nm and 663 nm via spectrophotometry, and the content of chlorophyll a and b in the leaves was calculated according to the formula (Porra et al., 1989).

After harvesting, the maize plants were divided into roots and shoots. These parts were initially subjected to 105°C for 30 min in an oven, then dried to a constant weight at 75°C, with their biomasses subsequently recorded.

### 2.5 Determination of the mycorrhizal colonization rate, spore number, and glomalin-related soil protein (GRSP) content

After the maize plants were harvested on Day 45, the soil adhering to the maize roots was collected as the rhizosphere soil. After the rhizosphere soil was kept indoors for 7 days and air-dried, a 20.0 g sample was weighed. Then, the AMF spores were separated by the wet sieving decantation-sucrose centrifugation method (Daniels and Skipper, 1982). The AMF spore number was counted via a stereomicroscope, and the number was calculated.

Moreover, 1.0 g of air-dried soil was weighed and placed into a centrifuge tube. The total glomalin-related soil protein (T-GRSP) was isolated from the soil using 8 mL of 50 mmol/L sodium citrate buffer (pH 8.0) at 121°C for 90 min, followed by centrifugation at 10,000 rpm for 6 min. This process was repeated 5 times. Similarly, the easily extractable glomalin-related soil protein (EE-GRSP) was obtained by treating the soil with 8 mL of 20 mmol/L citric acid buffer (pH 7.0) at 121°C for 30 min and centrifuging at 10,000 rpm for 6 min, also repeated 5 times. Finally, the supernatants were centrifuged again at 10,000 rpm for 10 min to obtain a new supernatant. Then, the concentrations of T-GRSP and EE-GRSP in the supernatant were determined by the Coomassie brilliant blue method, and the contents of T-GRSP and EE-GRSP in the rhizosphere soil were calculated (Wright and Upadhyaya, 1998).

After the maize roots were separated and cleaned thoroughly, some fibrous roots were sampled randomly and cut to approximately 1 cm in length for at least 80 segments. Then, these root segments were transferred into a tube with 10% (weight/volume) potassium hydroxide solution for dissociation at 90°C for 1 h in a water bath. At least 50 root segments were picked and placed on glass slides for staining with 0.1% acid fuchsin for 30 min and then destained with 50% lactoglycerin for 48 h. AMF colonization was observed under a microscope (Leica DM2000 LED, Leica Microsystems, Wetzlar, Germany), and the AMF colonization rate was calculated by the cross method (McGonigle et al., 1990).

### 2.6 Determination of cadmium contents in maize

Maize roots and shoots samples were crushed into powder smaller than 0.25 mm. A powder sample of 0.5 g was digested in 6 mL of nitric acid and 2 mL of perchloric acid into a transparent digestive liquid according to the wet digestion method and transferred into a volumetric bottle to a volume of 50 mL with distilled water. The Cd concentration in the solution was determined by a flame atomic absorption spectrophotometer (ICE-3000-SERIESThermo Scientific, Franklin, MA, USA). Then, the Cd content in maize was calculated according to the relevant formula. Cd uptake in maize (μg/plant) = biomass of maize (g/plant) × Cd content in maize (mg/kg).

### 2.7 Determination of cadmium availability and speciation in soil

Five grams of air-dried rhizosphere soil was weighed and placed into an Erlenmeyer flask (100 mL) filled with 0.01 mM CaCl_2_ solution (25 mL). Then, the flask was shaken at 120 revolutions per minute (rpm) for 120 min and filtered using quantitative filter paper into a volumetric flask to a volume of 50 mL with distilled water. Finally, the Cd concentration in solution was determined by a flame atomic absorption spectrophotometer, and the Cd availability (CaCl_2_-extracted) was calculated according to the relevant formula (Bao, 2000).

A 1.0 g of air-dried soil was used to determine the Cd speciation according to the Community Bureau of Reference (BCR) extraction method (Quevauviller et al., 1994). A 1.0 g soil sample was shaken at 22°C for 16 h with 40 mL of a 0.11 mol/L acetic acid (HAc) solution and centrifuged at 3000 rpm for 20 min. The supernatant was used to determine the acid-extractable Cd content. The residue was extracted with 40 mL of 0.5 mol/L NH_2_HCl solution at 22°C for 16 h and centrifuged at 3000 rpm for 20 min. The supernatant was used to determine the reducible Cd content. The residue was shaken with 10 mL of 8.8 mol of H_2_O_2_ at 22°C for 1 h and digested at 85°C to a volume of 1 mL. Then, 50 mL of 1.0 mol/L NH_4_Ac solution was added, and the mixture was oscillated at 22°C for 16 h. The mixture was subsequently centrifuged at 3000 rpm for 20 min, after which the supernatant was collected for determination of the oxidizable Cd content. After the above steps, 0.10 g of residue was weighed, 3 mL of HCl, 2 mL of HNO_3_, 1 mL of HClO_4_, and 5 mL of hydrofluoric acid (HF) solution were added. The residue was digested using the wet digestion method to yield a transparent digest for assessing the residual Cd content.

### 2.8 Determination of cadmium concentration and form in soil solution and leachate

The pH values of the soil solution and leachate were determined by a pH meter. Following filtration, 5 mL of the filtrate underwent digestion with 5 mL of HNO_3_ and 5 mL of H_2_O_2_ at 90–95°C on an electric heating plate. The digestion mixture was transferred to a volumetric flask to a volume of 50 mL with distilled water. The Cd concentration was determined by a graphite furnace atomic absorption spectrophotometer (ICE 3000 Series, Thermo Scientific, Franklin, MA, USA), and then the Cd concentrations in the soil solution and leachate were calculated. Cd leaching loss (μg) = Cd concentration (μg/mL) × volume of leachate (mL).

Ion chromatography was used to determine the ions in the leachate and soil (Michalski, 2006), and the instrument used was an Aquion instrument (Thermo Fisher Scientific, Waltham, MA, United States). The anions used an AG19 chromatographic column and an AS19 protective column equipped with a KOH automatic elution device (concentration of 20 mM), and the flow rate was 1 mL/min to determine the ion concentrations of SO_4_^2−^, Cl^−^, and F^−^. To determine the ion concentrations of Na^+^, Mg^2+^, Ca^2+^, and NH_4_^+^, an AG12 chromatographic column, AS12 protective column, and methylsulfonic acid eluent (concentration of 20 mM) were used, and the flow rate was 1 mL/min.

The pH, cation content, anion content, and cadmium content were recorded with visual MINTEQ3.1 software (Environmental Protection Agency, United States) to calculate the occurrence of cadmium in the soil solution and leachate, including Cd^2+^, CdSO_4_ (aq), CdOH^+^, CdF^+^, and CdCl^+^.

### 2.9 Data analysis

The experimental data were the average of 5 replicates. IBM SPSS Statistics 21 was used for significance analysis and correlation analysis of the data. The mean value and standard error of the data were analyzed by one-way ANOVA. Differences were tested by Duncan’s multiple comparison test. The significance level was set at *p* < 0.05. The Pearson correlation coefficient was used to analyze the correlation, Origin 2022 was used for chart drawing, and IBM SPSS Amos 26 was used to construct the structural equation model.

## 3 Results

### 3.1 Characteristics of ERM infestation in maize roots

Maize root infection rates ranged from 20.7 to 39.6% when inoculated with AMF, with spore counts per gram of soil ranging from 10.5 to 18.3, indicating that AMF effectively infected maize roots.

Compared with the static treatment, the rotation treatment caused a decrease in the content of easily extracted glomalin-related soil protein (EE-GRSP) inside and outside the in-growth core from 1.15 and 0.96 mg/g to 0.85 and 0.81 mg/g, a decrease of 16 and 5%, respectively, but had no significant effect on the total glomalin-related soil protein (T-GRSP) content (Supplementary Figure S2).

### 3.2 Effect of ERM on maize plant height, biomass, and photosynthetic physiology

Compared with the static treatment, the rotation treatment reduced the plant height of maize from 32.54 cm to 27.52 cm, with a decrease of 15%. But had no significant effect on the maize total biomass or the shoot or root biomass.

Compared with the static treatment, the rotation treatment caused a significant decrease in the chlorophyll b content and net photosynthetic rate of maize leaves, by 30 and 43%, respectively, while the transpiration rate increased by 51% (Table 1).

**Table 1:** Effects of static and rotated treatments on photosynthetic physiology of maize.

Treatment	Photosynthetic rate (μmol CO_2_·m^−2^·s^−1^)	Intercellular CO_2_ concentration (μmol·mol^−1^)	Transpiration rate (mmol H_2_O·m^−2^·s^−1^)	Chlorophyll a Content (mg·g^−1^)	Chlorophyll b Content (mg·g^−1^)
Static	7.31 ± 1.42**	243.2 ± 26.02	2.16 ± 0.2	2.55 ± 0.34	2.27 ± 0.1**
Rotated	4.19 ± 0.41	225 ± 23.82	3.25 ± 0.45**	2.10 ± 0.35	1.58 ± 0.25

All values represent the mean ± error (SE), *n* = 5. “**” indicates highly significant correlation at p < 0.01 level.

### 3.3 Effect of ERM on cd content and uptake in maize

**Figure 1:**
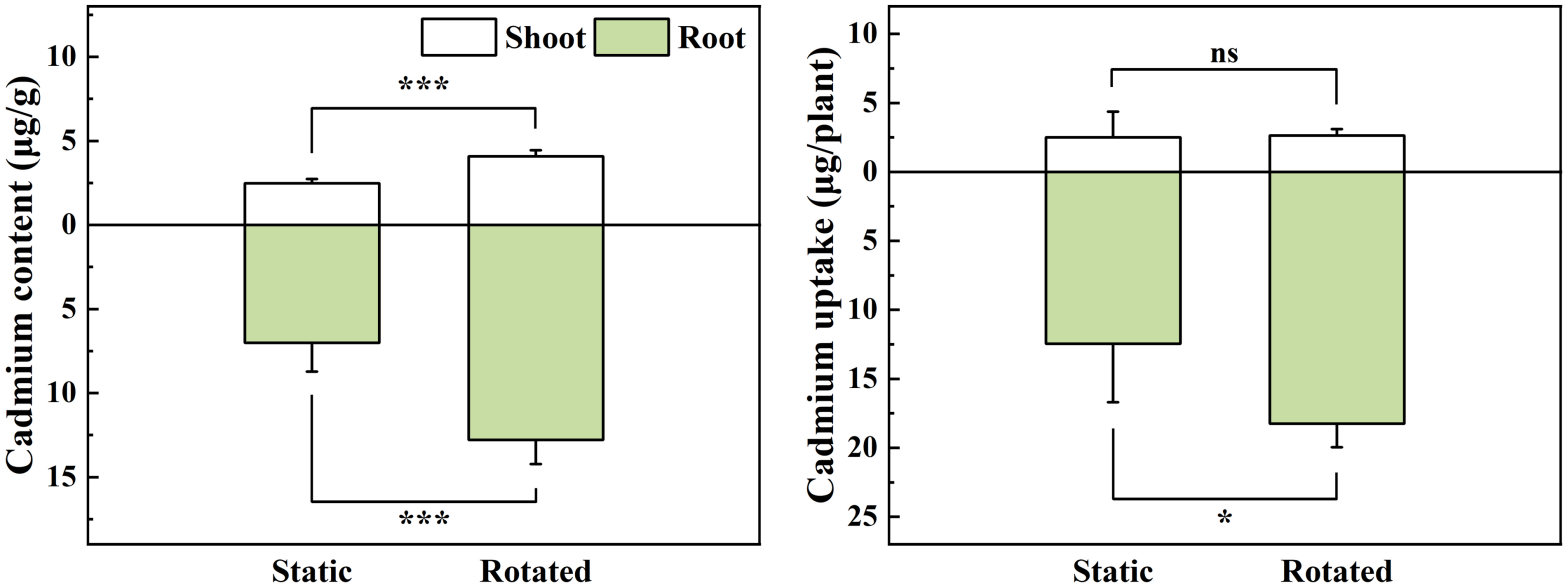
Effects of static and rotated treatments on **(A)** Cd content and **(B)** uptake in maize. All values represent the mean ± error (SE), *n* = 5. “*” indicates significant correlation at *p* < 0.05 level, “***” indicates highly significant correlation at *p* < 0.001 level; “ns” indicate no significant differences between treatments.

Compared with the static treatment, the rotation treatment caused a significant increase in the Cd content in the shoot and root of maize, by 64 and 82%, respectively (Figure 1A), while it increased the Cd uptake in the root by 46% (Figure 1B).

### 3.4 Effects of ERM on the form and CaCl_2_-extraction of cd in soil

**Figure 2:**
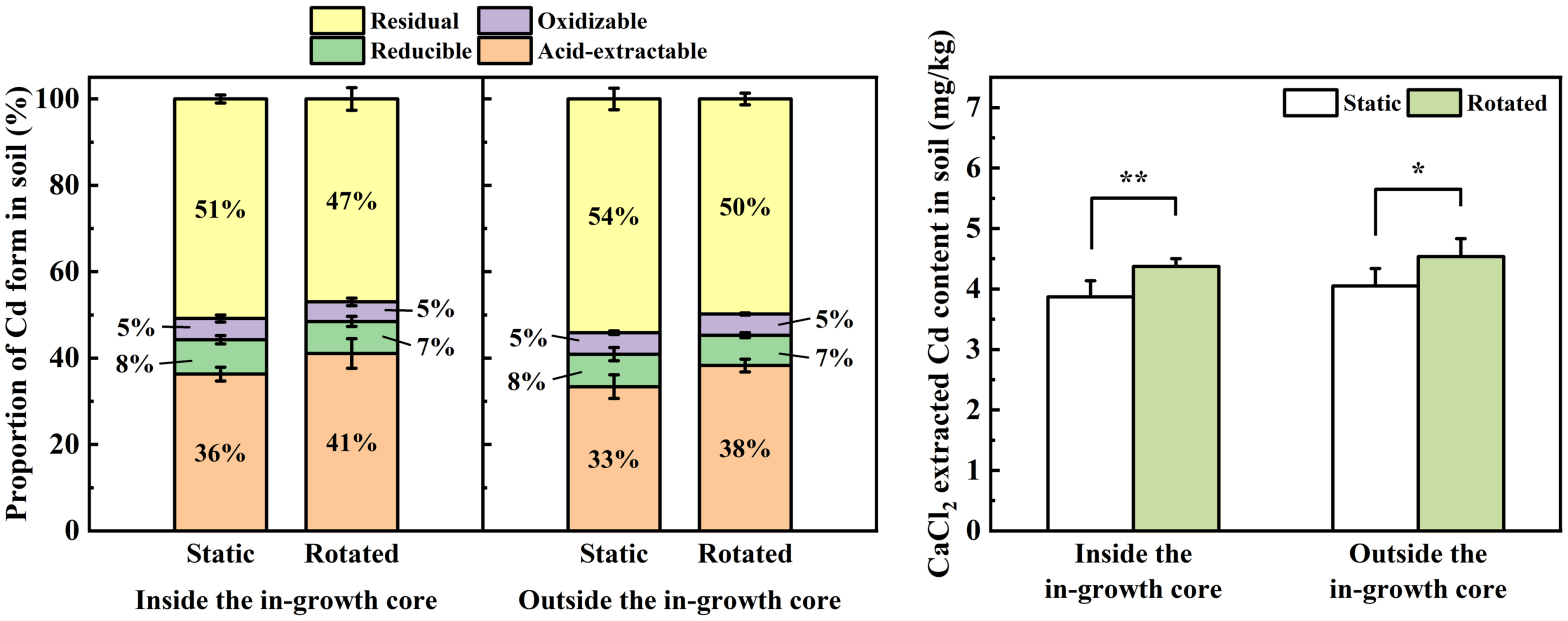
Effects of static and rotated treatments on **(A)** form and **(B)** CaCl_2_-extracted Cd in soil. All values represent the mean ± error (SE), *n* = 5. “*” indicates significant correlation at *p* < 0.05 level, “**” indicates highly significant correlation at *p* < 0.01 level.

Compared with the static treatment, the rotation treatment caused a significant increase in the proportion of acid-extractable Cd in the rhizosphere soil of maize by 13%, a significant decrease in the proportion of residual Cd by 8%, a significant increase in the proportion of acid-extractable Cd in the non-rhizosphere soil by 15%, and a significant decrease in the proportion of residual Cd by 8% (Figure 2A).

Compared with the static treatment, the rotation treatment caused a significant increase in the CaCl_2_-extracted Cd content in the rhizosphere and non-rhizosphere soils by 13 and 12%, respectively (Figure 2B).

### 3.5 Effect of AMF on soil solution cd concentration and leaching loss

**Figure 3:**
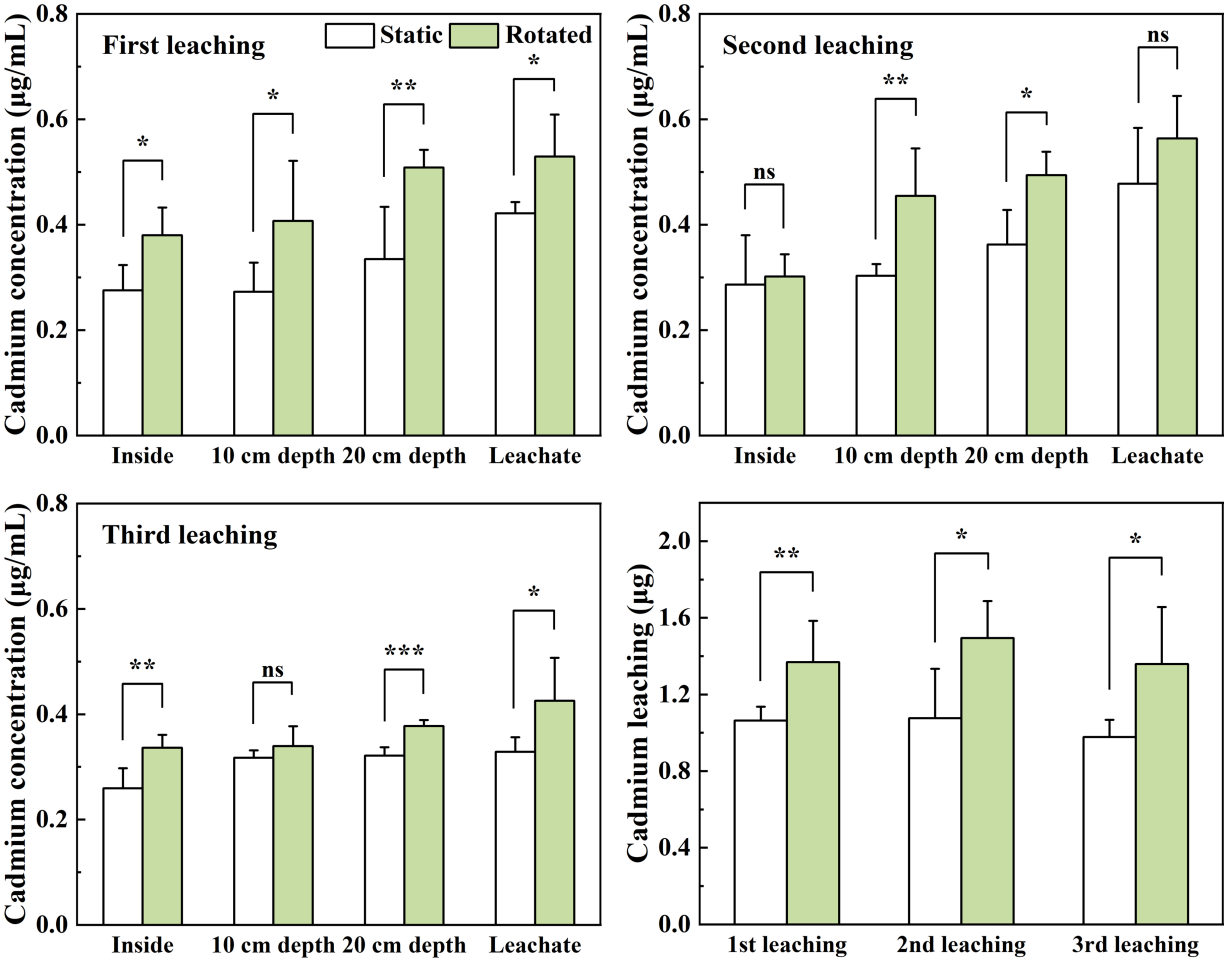
Effects of static and rotated treatments on **(A)** Cd concentration and **(B)** leaching loss from soil solution and leachate. All values represent the mean ± error (SE), *n* = 5. “*” indicates significant correlation at *p* < 0.05 level, “**” indicates highly significant correlation at *p* < 0.01 level, “***” indicates highly significant correlation at *p* < 0.001 level; “ns” indicate no significant differences between treatments.

Compared with the static treatment, the rotation treatment caused a significant increase in the Cd concentration in the soil solution within the growing core, the 10 cm depth soil solution, the 20 cm depth soil solution, and the leachate by 38, 49, 52, and 26%, respectively, under the first leaching; the Cd concentrations in the 10 cm and 20 cm depth soil solutions increased by 50 and 36%, respectively, under the second leaching; and the Cd concentrations in the soil solution in the in-growth core, 20 cm depth soil solution, and leachate under the third leaching increased by 30, 17, and 30%, respectively (Figures 3A–C).

The rotation treatment caused a significant increase in the leaching loss of Cd by 29, 39, and 39%, respectively, in the three leaching tests (Figure 3D).

### 3.6 Effects of ERM on the form of cd in soil solution and leachate

In the three leaching tests, Cd^2+^ predominated, comprising 93.04 to 99.04% of the concentration; CdSO_4_ (aq) ranked next, at 0.71 to 6.68%; while CdF^+^ was least, ranging from 0.02 to 0.07%.

**Figure 4:**
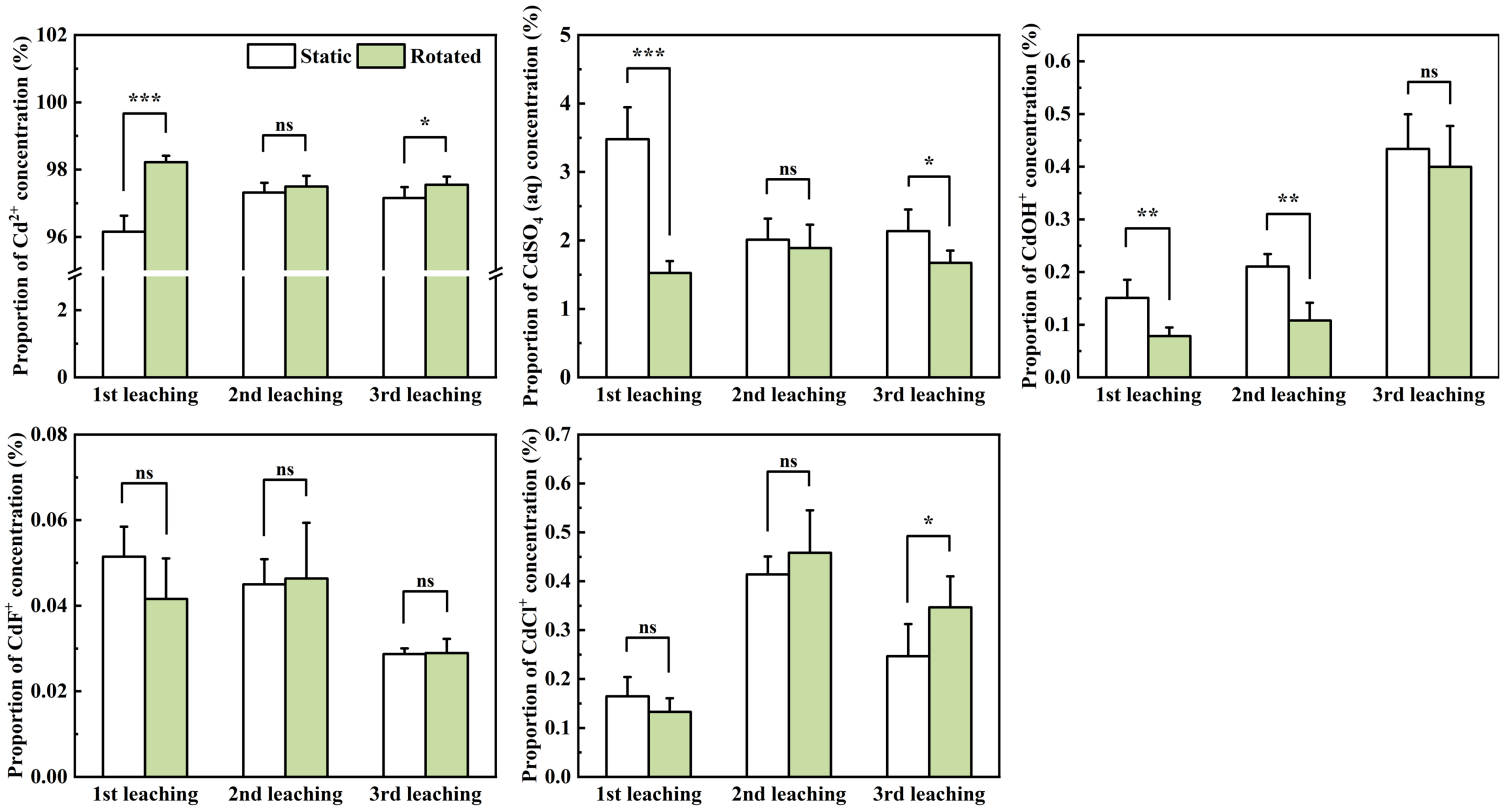
Effects of static and rotated treatments on the form of Cd in soil solution within the in-growth core. All values represent the mean ± error (SE), *n* = 5. “*” indicates significant correlation at *p* < 0.05 level, “**” indicates highly significant correlation at *p* < 0.01 level, “***” indicates highly significant correlation at *p* < 0.001 level; “ns” indicate no significant differences between treatments.

Within in-growth cores, compared with the static treatment, rotational treatment induced significant variations in the proportions of different Cd ion forms during leaching. Specifically, Cd^2+^ proportion increased by 2.1% during the first leaching, while CdSO_4_(aq) proportion decreased by 56.1 and 6.1% during the first and third leaching, respectively. Moreover, the CdOH^+^ proportion decreased by 47.9 and 48.7% during the first and second leaching, and the CdCl^+^ proportion increased by 40.5% during the third leaching (Figure 4).

**Figure 5:**
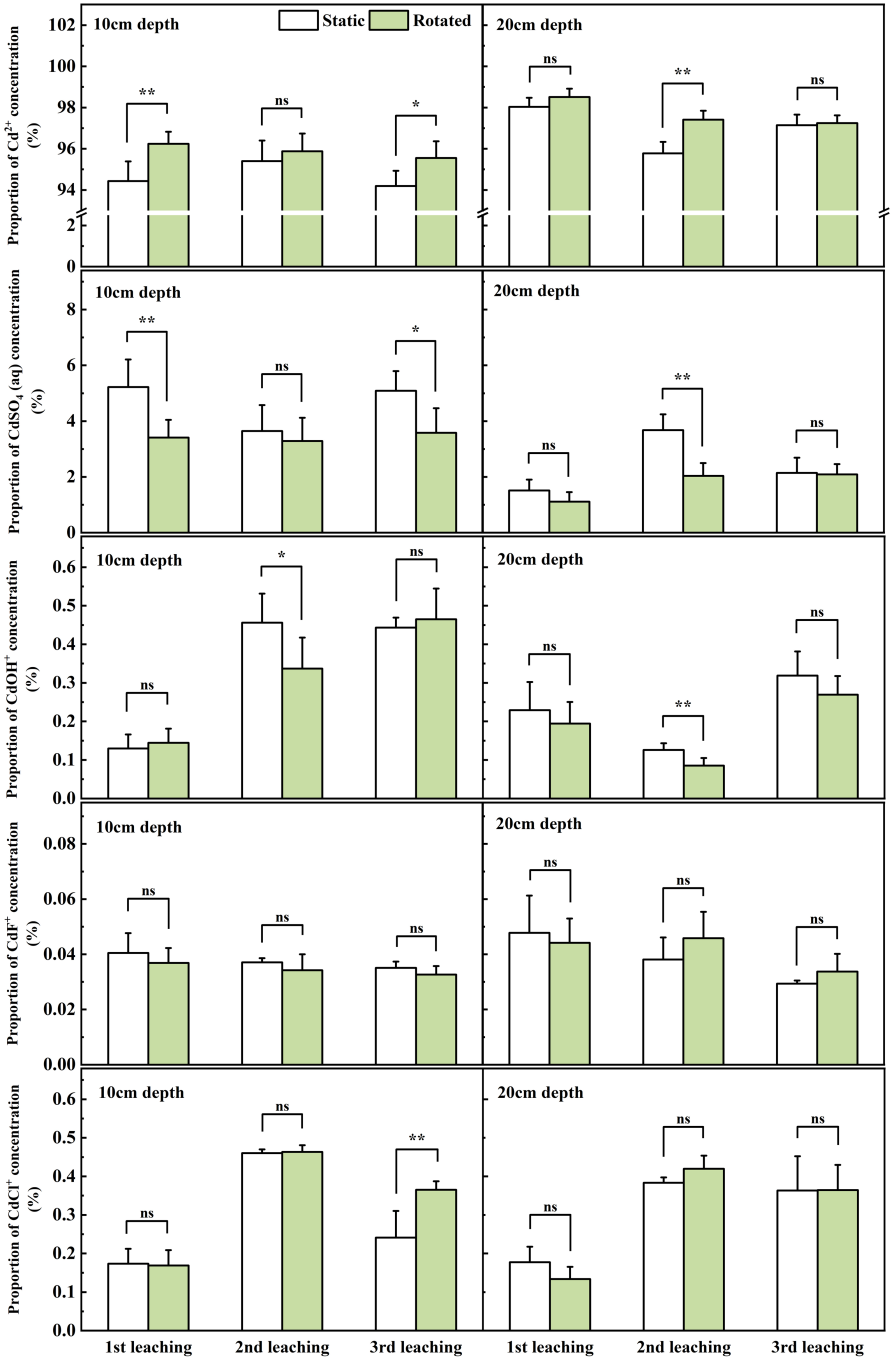
Effects of static and rotated treatments on the form of Cd in soil solution outside the in-growth core. All values represent the mean ± error (SE), *n* = 5. “*” indicates significant correlation at *p* < 0.05 level, “**” indicates highly significant correlation at *p* < 0.01 level; “ns” indicate no significant differences between treatments.

At the sampling point at 10 cm depth outside the in-growing core, rotational treatment caused a 1.9% increase in Cd^2+^ proportion during the first leaching, a decrease in CdSO_4_(aq) proportion by 34.7 and 29.6% during the first and third leaching, a 26.1% decrease in CdOH^+^ proportion during the second leaching, and a 51.4% increase in CdCl^+^ proportion during the third leaching (Figure 5).

At the sampling point at the 20 cm depth outside the in-growth core, rotational treatment caused a 1.7% increase in Cd^2+^ proportion during the second leaching, a 44.5% decrease in CdSO_4_(aq) proportion during the second leaching, and a 32.1% decrease in CdOH^+^ proportion during the second leaching (Figure 5).

**Figure 6:**
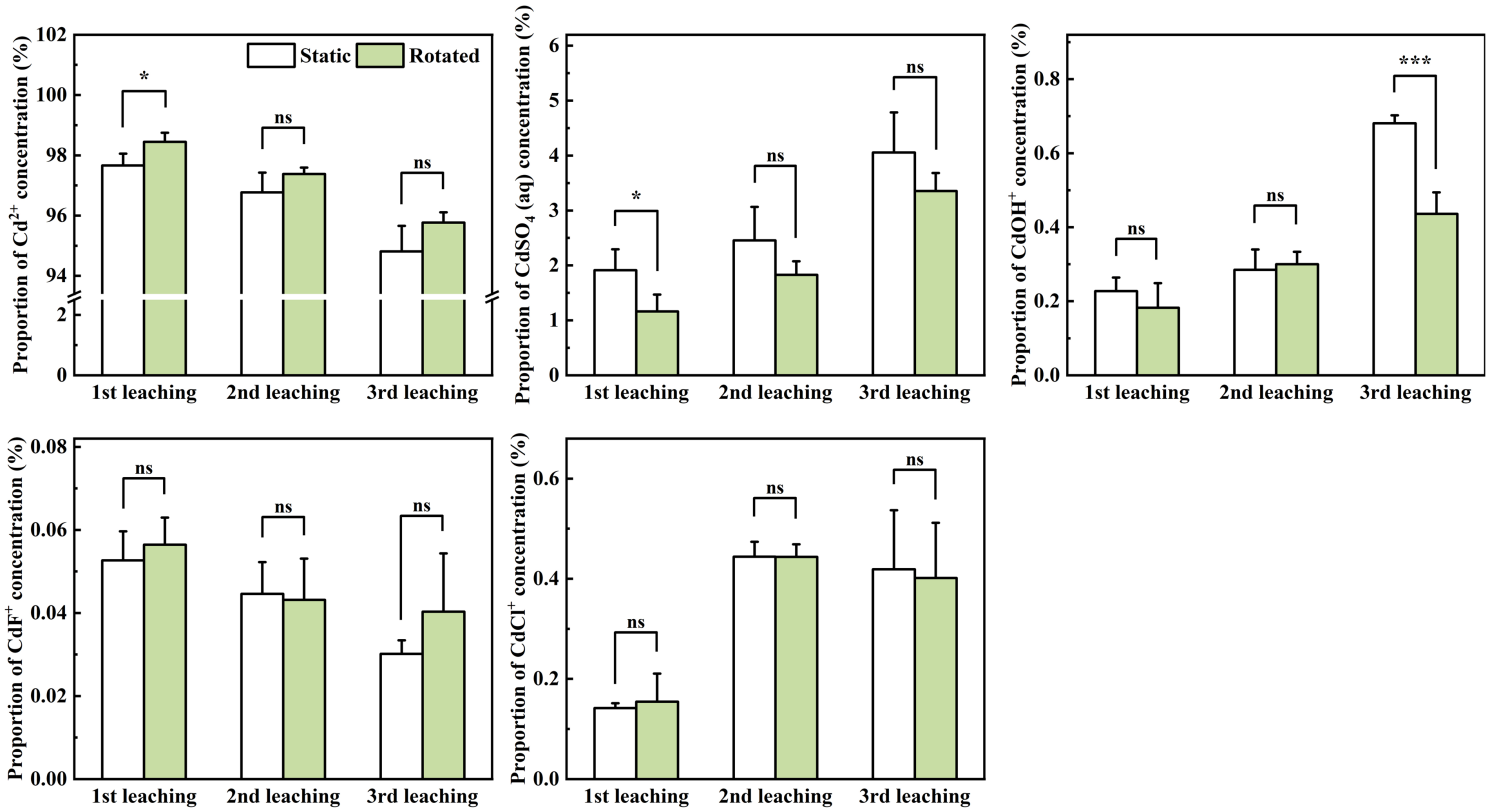
Effects of static and rotated treatments on the form of Cd in the leachate. All values represent the mean ± error (SE), *n* = 5. “*” indicates significant correlation at *p* < 0.05 level, “***” indicates highly significant correlation at *p* < 0.001 level; “ns” indicate no significant differences between treatments.

In the leachate, rotational treatment caused a 0.8% increase in Cd^2+^ proportion during the first leaching, a 39.3% decrease in CdSO_4_(aq) proportion during the first leaching, and a 36.0% decrease in CdOH^+^ proportion during the third leaching (Figure 6).

### 3.7 Analysis of the effect of ERM on the migration of cd from contaminated soil

**Figure 7:**
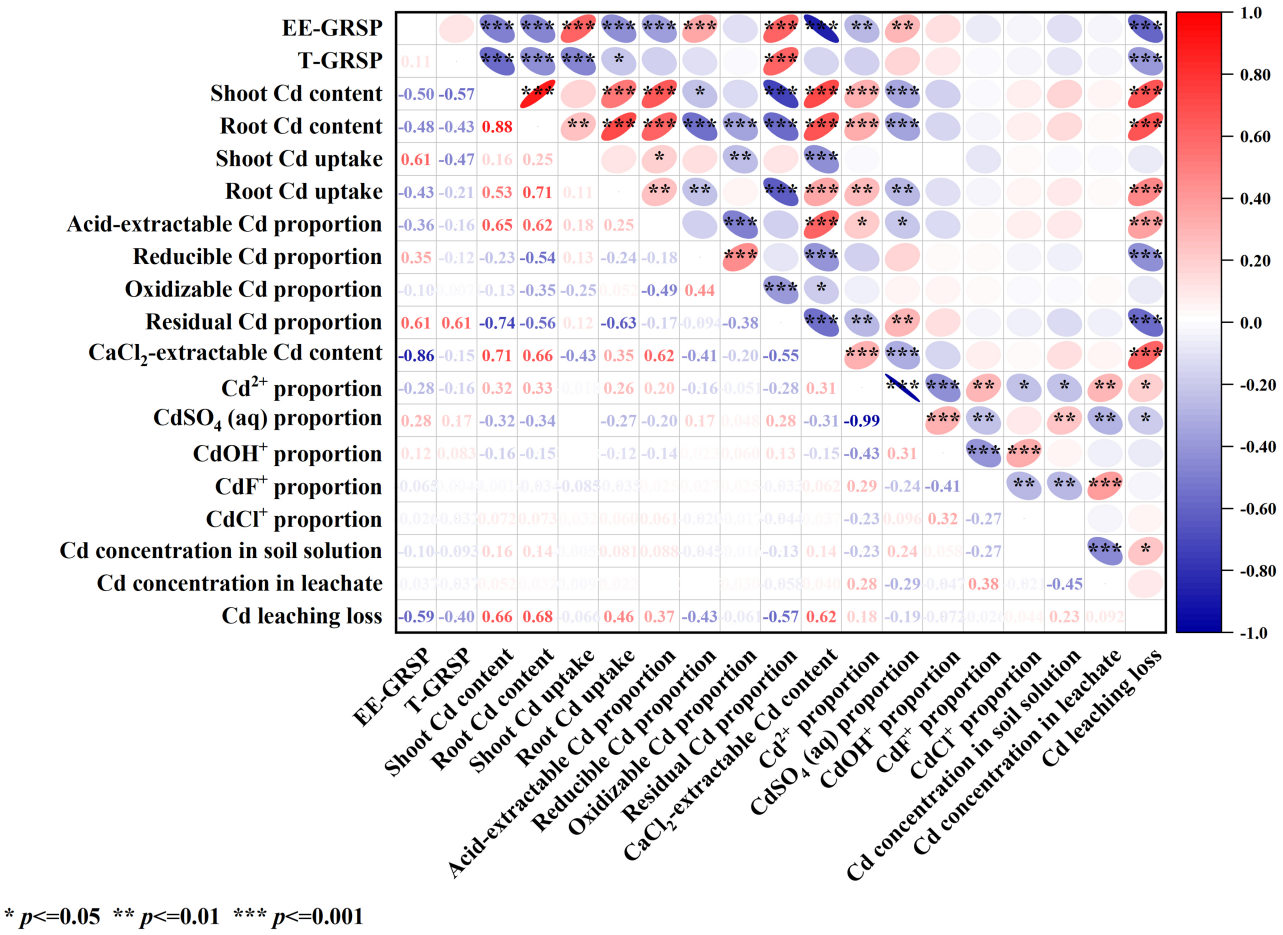
Correlation analysis of the effects of AMF on the morphology and migration of Cd in contaminated soil-maize.

Correlation analysis (Figure 7) revealed that the contents of EE-GRSP and T-GRSP in contaminated soil were significantly negatively correlated with Cd leaching loss (*p* < 0.001) and were significantly (*p* < 0.001) or significantly (*p* < 0.05) positively correlated with the Cd content in shoots and roots and with Cd uptake in roots. These results indicate that GRSP secreted by AMF through its mycelium helped reduce Cd leaching loss in contaminated soil and Cd uptake by plants.

The content of EE-GRSP in contaminated soil was significantly negatively correlated with the content of acid-extractable Cd, CaCl_2_-extractable Cd, and solution Cd^2+^ in soil (*p* < 0.01) and significantly positively correlated with the content of reducible Cd, residual Cd and solution CdSO_4_ (aq) in soil (*p* < 0.01). GRSP secreted by AMF mycelium reduces the content of Cd^2+^ in solution, acid-extractable Cd and CaCl_2_-extractable Cd in soil, and increases the content of residual Cd in soil and CdSO_4_ (aq) in solution.

The acid-extractable Cd, CaCl_2_-extractable Cd, and solution-extractable Cd^2+^ in contaminated soil were significantly (*p* < 0.05) or extremely significantly (*p* < 0.01) positively correlated with the Cd content in shoots and roots, Cd uptake in roots, and Cd leaching loss. The contents of reducible Cd, residual Cd, and solution CdSO_4_ (aq) in contaminated soil were significantly (*p* < 0.05) or extremely significantly (*p* < 0.01) negatively correlated with the Cd content in the shoot and root and with the Cd uptake in the root. These results indicate that the reducible and residual Cd in soil and the solution of CdSO_4_ (aq) helped reduce plant Cd uptake and Cd leaching loss in contaminated soil.

**Figure 8:**
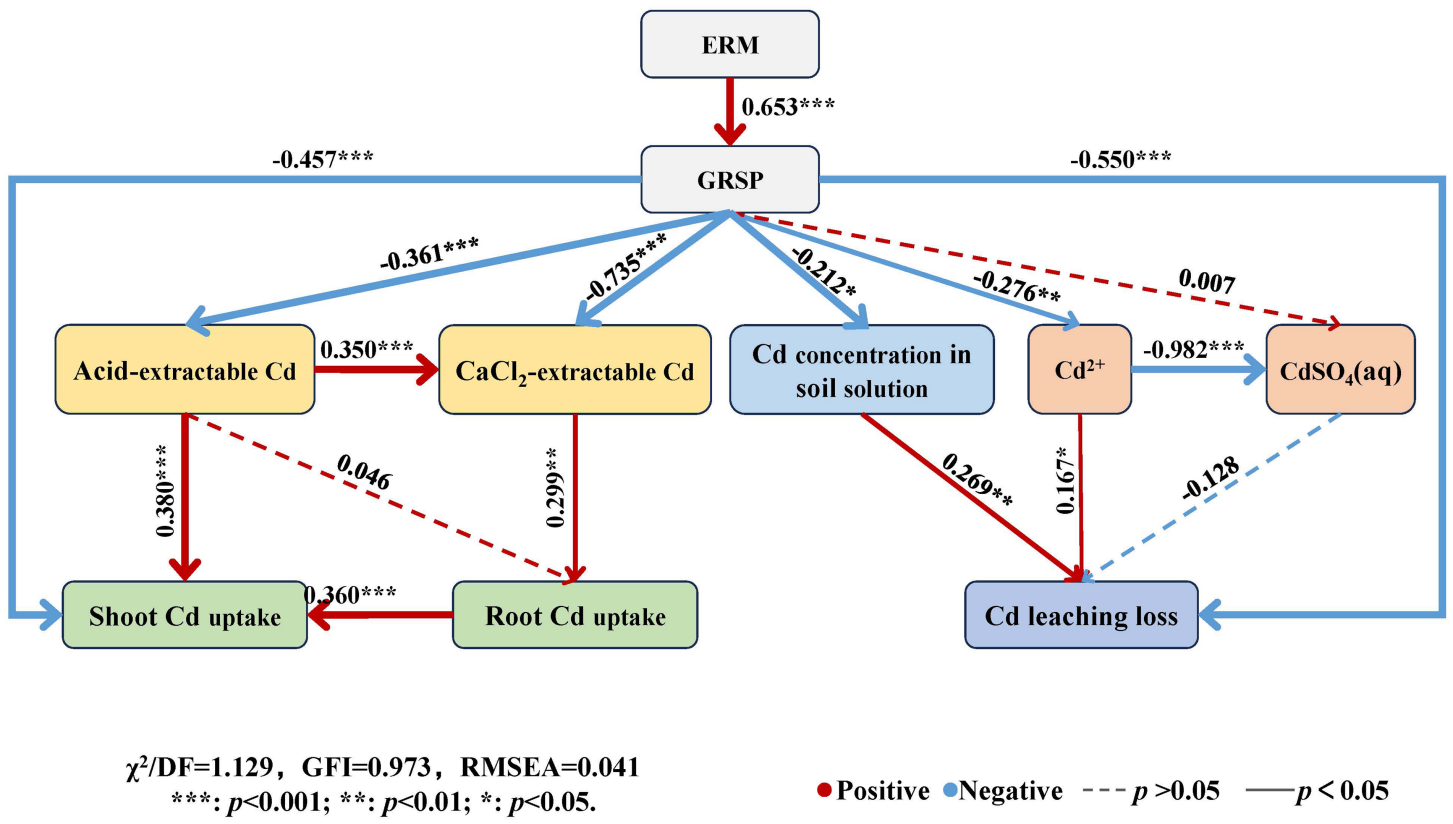
Structural equation model of influencing factors of Cd migration in contaminated soil. Note: The red line indicates a positive relationship, the blue line indicates a negative relationship, and the path with insignificant coefficients is represented by a dotted line. The online numbers represent the total effect of standardization.

The structural equation model (Figure 8) showed that the path coefficient of ERM to GRSP was 0.653 (*p* < 0.001), and the path coefficients of GRSP to acid-extractable Cd, CaCl_2_-extractable Cd, and shoot Cd uptake were − 0.361 (*p* < 0.001), −0.735 (*p* < 0.001), and − 0.457 (*p* < 0.001), respectively, indicating that GRSP directly had a significant or very significant negative effect on available Cd in soil and maize Cd uptake. Moreover, the path coefficients of the GRSP to the Cd concentration in the soil solution and the free Cd^2+^ and Cd leaching losses were − 0.212 (*p* < 0.05), −0.276 (*p* < 0.01) and − 0.550 (*p* < 0.001), respectively, indicating that the GRSP directly reduced the Cd concentration, the proportion of free Cd^2+^ in the soil solution and its leaching loss.

Moreover, the path coefficient of acid-extractable Cd and shoot Cd uptake was 0.380 (*p* < 0.001), and that of CaCl_2_-extractable Cd in soil to root Cd uptake was 0.299 (*p* < 0.01), indicating that the available Cd in soil had a very significant positive effect on maize Cd uptake. The path coefficients of the Cd concentration and free Cd^2+^ in the soil solution for Cd leaching loss were 0.269 (*p* < 0.01) and 0.167 (*p* < 0.05), respectively, indicating that the Cd concentration in the soil solution contributed to Cd leaching loss. Thus, AMF can directly and indirectly reduce Cd uptake by plants and Cd leaching loss by decreasing Cd availability in soil and soil solution through the GRSP secreted by ERM.

## 4 Discussion

AMF, as soil fungi that can colonize roots and form symbiotic relationships with host plants, have a remarkable impact on plant growth and performance (Yang et al., 2015). AMF establishes a symbiotic relationship with plant roots, enhancing root surface area, facilitating nutrient absorption and transport, and improving nutrient utilization efficiency, thus promoting photosynthesis and growth of the plant (Song et al., 2021; Bhantana et al., 2021). ERM helps to increase the plant’s ability to absorb and retain water, maintain the water balance in the plant, and promote photosynthesis, growth, and development (Begum et al., 2019). Additionally, ERM can supply a portion of the plant’s carbon needs, promoting the accumulation and distribution of photosynthetic products within the plant, thereby augmenting growth and metabolic functions (Wahab et al., 2023). Together, these mechanisms optimize nutrient absorption, water regulation, and carbon supply in plants, ultimately advancing plant growth and development. Disruption of the ERM hampers the functionality of AMF, leading to detrimental impacts on plant growth (Bahadur et al., 2019). In the present study, compared with the static treatment with an undisrupted ERM, the rotation treatment to disrupt the ERM outside the in-growth core caused significant decreases in the plant height and photosynthetic physiology of the maize plants. These findings indirectly underscore the beneficial role of ERM in promoting maize growth.

Under abiotic stresses such as Cd pollution in soil, the presence of AMF enhanced plant resistance to Cd stress with the help of ERM in soil (Wu et al., 2014; Bargaz et al., 2018). First, ERM reduced the amount of Cd entering plant roots through adsorption, thereby reducing Cd toxicity to plants (Pan et al., 2023). In addition, the ERM produced a special glycoprotein, glomalin-related soil protein (GRSP), which is secreted on the surface of the extraradical mycelium of symbionts. GRSP can bind or chelate heavy metals and therefore plays an important role in fixing heavy metal ions such as Cd in soil (Gujre et al., 2021; Wang et al., 2012). Thus, GRSP contributed to reducing Cd bioavailability in soil and Cd uptake by plants (Xiao et al., 2021; Li et al., 2018). In the present study, compared with the static treatment, the rotation treatment caused significant increases in the Cd content in the shoots and roots of maize plants grown in the in-growth core. The structural equation model (SEM) also indicated that the GRSP was a key factor that was negatively correlated with the Cd content in maize. Therefore, the present study also confirmed that ERM secreted GRSP to inhibit Cd availability and uptake by plants.

Many studies have shown that AMF immobilizes Cd in soil through ERM and mycorrhizal structures, which convert active Cd ions into inactive species and reduce Cd bioavailability in soil (Fang et al., 2024; Zhang et al., 2019). The GRSP secreted by ERM immobilizes various heavy metals, including Cd, Pb, Cu, etc., and plays an important role in alleviating the bioavailability and toxicity of heavy metals in soil (Ai et al., 2022). In the present study, the rotation treatment increased the proportion of acid-extractable Cd and reduced the proportion of residual Cd in the soil, and the CaCl_2_-extracted Cd content was significantly greater in the rotation treatment than in the static treatment. These results indicated that ERM disruption resulted in the transformation of Cd speciation from a low activity (residual) to a higher activity (acid-extractable), and ERM reduced the CaCl_2_-extracted Cd content in the soil. The rotation of the in-growth core to break the ERM outside led to a decrease in the GRSP content in the soil.

Additionally, previous studies have shown that the GRSP secreted by ERM has a strong adsorption capacity for Cd ions and effectively adsorbs 76% of Cd ions in water (Wang et al., 2019; Yuan et al., 2022; Zhan et al., 2023). Therefore, GRSP significantly reduced the solubility and migration of Cd ions from water. In the present study, the ERM disruption in the Rotated treatment caused significant increases in the Cd concentration in the soil solution, indirectly indicating that the ERM significantly limited the bioavailability and migration of Cd ions in the soil solution. Therefore, the rotation treatment significantly increased Cd leaching loss from the soil, indicating that ERM plays a functional role in inhibiting Cd leaching loss.

Furthermore, ERM growth and nutrient absorption from soil transform the chemical composition and forms of Cd ions in the soil solution (Ullah et al., 2023). For example, ERM can absorb nutrients such as nitrogen, phosphorus, potassium, calcium, and magnesium from the soil solution, resulting in a change in the chemical properties of the soil solution (Fall et al., 2022). Studies have shown that ERM can affect the forms of Cd ions in soil solution through adsorption, chelation, and precipitation (Zhang et al., 2021). On the one hand, the surface of ERM is negatively charged, and these negative charges can interact with the positive charges of Cd ions so that Cd ions are adsorbed on the surface of the mycelium (Han et al., 2021). This adsorption can reduce the concentration of free Cd ions in soil solution, thereby reducing its toxicity and bioavailability. On the other hand, ERM also secretes some organic substances, such as low molecular weight organic acids, into the soil solution and then promotes the transformation of active Cd ions into inactive forms, such as Cd oxides or Cd chelates, which helps to reduce the bioavailability and mobility of Cd ions. In addition, the substances secreted by ERM can react with Cd ions to form insoluble precipitates. These precipitates can cause Cd ions to exist in the soil solution in a precipitated state, reducing its dissolved concentration (Jiang et al., 2021; Wang et al., 2022). In the present study, the ERM disruption in the rotation treatment increased the proportion of Cd^2+^ ions and decreased the proportions of CdSO_4_(aq) and CdOH^+^ in the soil solution and leachate. Furthermore, the Cd^2+^ proportion in the soil solution was significantly positively correlated with Cd leaching loss, and the CdSO_4_(aq) proportion was significantly negatively correlated with Cd leaching loss. These results indirectly indicated that ERM contributed to the transformation of Cd^2+^ with greater activity to CdSO_4_ (aq) and CdOH^+^ with lower activity and to reducing Cd leaching loss from the soil solution.

Based on the in-growth core experiment, the present study indicated that ERM can potentially promote plant growth and inhibit Cd uptake by plants and Cd leaching loss by changing the Cd form and reducing the Cd concentration in the soil solution. This study aims to investigate the ERM in Cd contaminated soil by disrupting its growth. Understanding this role is crucial for comprehending the potential of ERM in remediating soil pollution. The experimental outcomes will serve as a foundation for crafting strategies to mitigate Cd contamination, enhancing soil quality and reducing crop toxicity. However, the effects of ERM on Cd migration in the field are still unclear. The mechanisms underlying the effects of ERM on the chemical properties of soil solutions are poorly understood. Therefore, more studies on the ecological effects of ERM on soil and soil solutions in the field should be conducted. Additionally, the mechanisms underlying the effects of ERM on soil properties and Cd migration in soil solutions should be further explored. Therefore, the potential functions of ERM should be further utilized in the ecological remediation and management of heavy metal-polluted soil.

## 5 Conclusion

Based on the in-growth-core experiment, the ERM disruption of the rotation treatment significantly reduced the photosynthetic physiology of leaves, and maize growth, and the GRSP content in the soil but increased the Cd availability in the soil, caused an increase in the percentage of acid-extractable Cd in the soil and a decrease in the percentage of residual Cd, which resulted in increases in the Cd content and uptake by maize. Additionally, ERM disruption increased the Cd concentration and the proportion of free Cd^2+^ in the soil solution, which caused an increase in Cd leaching loss from the soil. These results indirectly indicated that AMF plays a crucial role in inhibiting Cd uptake by plants and Cd leaching loss through the GRSP secreted by ERM in soil.

## Data Availability

The original contributions presented in the study are included in the article/Supplementary material, further inquiries can be directed to the corresponding authors.
