# Supplementary material for: Mycorrhizal extraradical mycelium can reduce cadmium uptake by maize and cadmium leaching from contaminated soil: based on an in-growth core experiment
Source: Front Microbiol. 2024 Dec 16;15:1507798. doi: 10.3389/fmicb.2024.1507798 (PMC11683564; doi:10.3389/fmicb.2024.1507798)
Supplement: Supplementary file 1 [file Data_Sheet_1.docx]

Supplementary Material

# Table S1 Basic physical and chemical properties of soil

| Index | Numerical value |
| --- | --- |
| pH | 6.98 |
| Organic matter(g/kg) | 42.74 |
| Total nitrogen(g/kg) | 1.33 |
| Total phosphorus(g/kg) | 1.06 |
| Total potassium(g/kg) | 11.96 |
| Alkaline hydrolysis nitrogen(g/kg) | 121.31 |
| Rapidly available phosphorus (mg/kg) | 86.52 |
| Rapidly available potassium(mg/kg) | 152.05 |
| Cadmium content(mg/kg) | 17.44 |


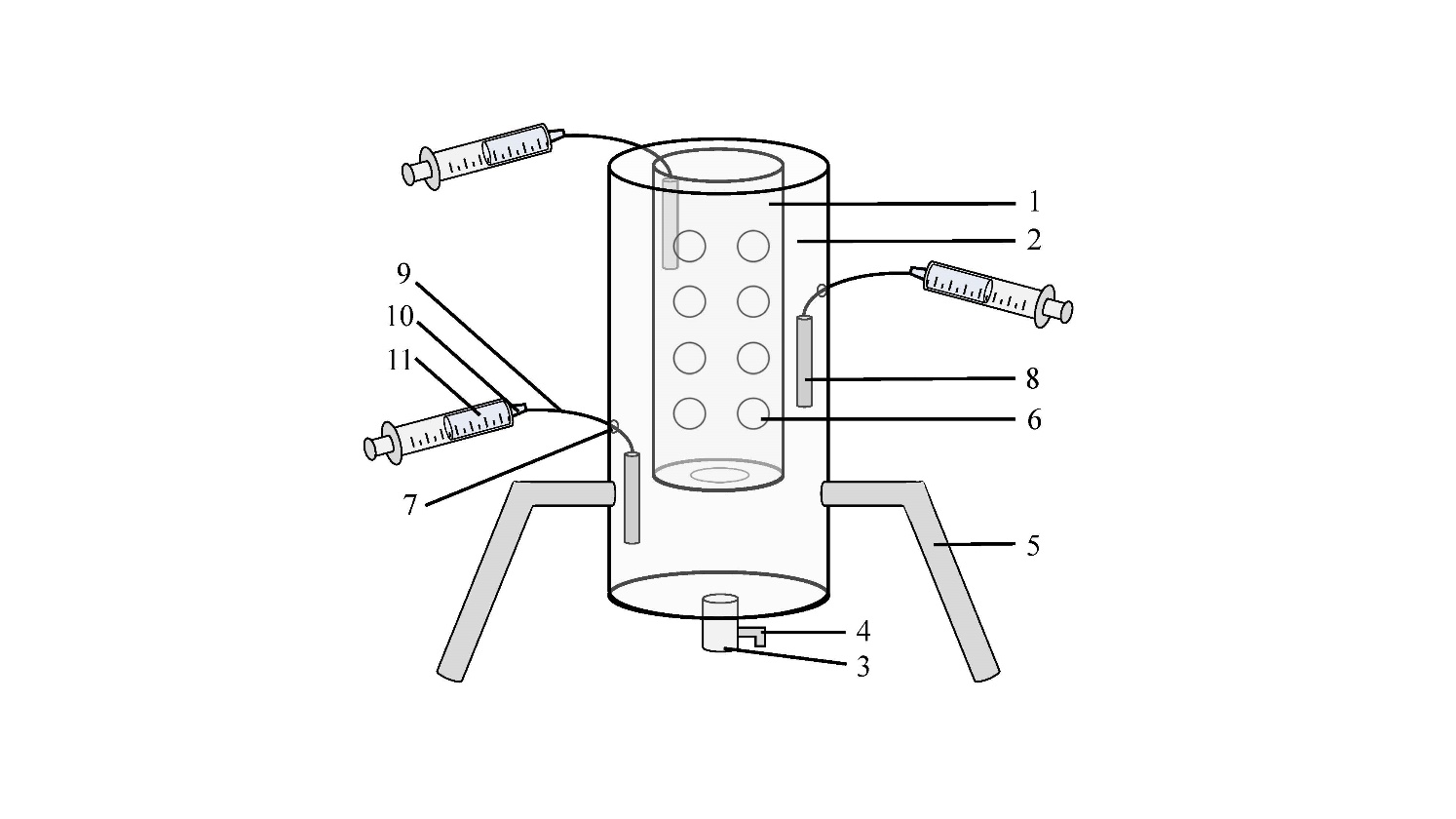


**Figure S1** Schematic diagram of the experimental device

Note: 1. in-growth core, 2. soil column, 3. drain pipe, 4. discharge cock, 5. holder, 6. foramen rotundum, 7. opening, 8. soil solution sampling head, 9. sampling hose, 10. rotating buckle, 11. needle-free injector.

**
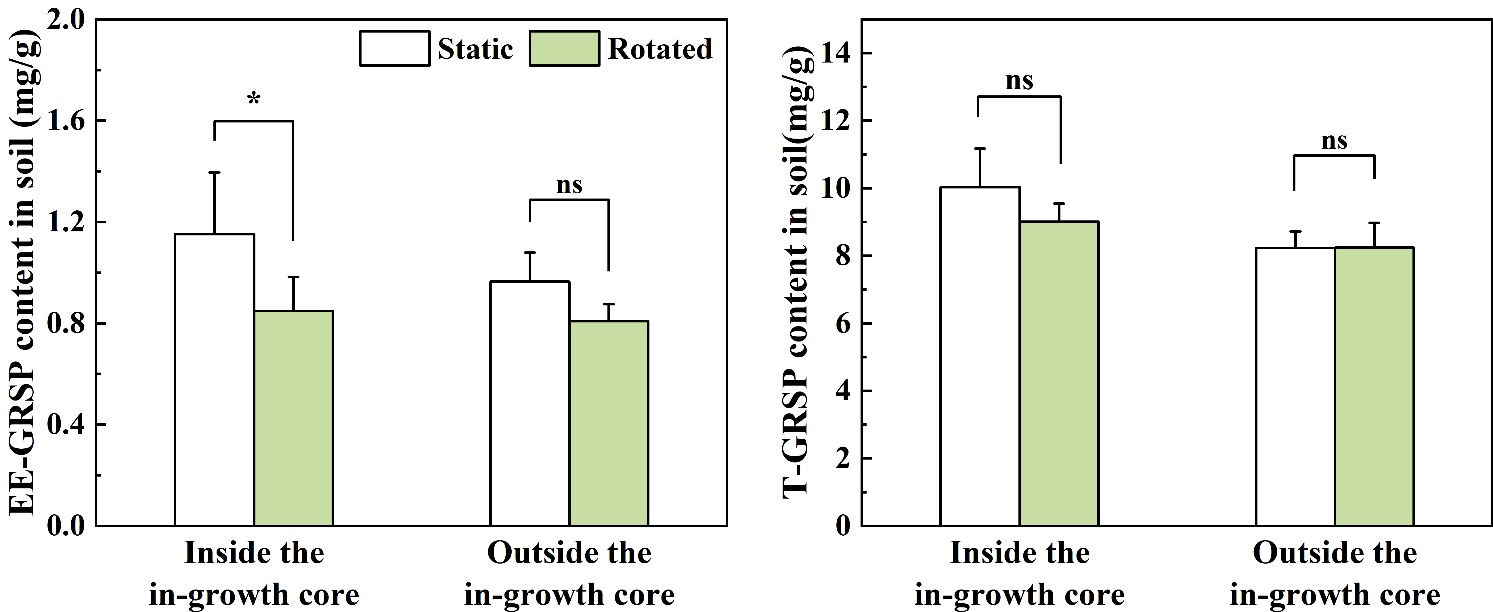
**

**Figure S2** Effects of static and rotated treatments on soil GRSP content

All values represent the mean±error (SE), n=5. “*” indicates significant correlation at *p*<0.05 level; “ns” indicate no significant differences between treatments.
